# The performance of sonographic antenatal birth weight assessment assisted with artificial intelligence compared to that of manual examiners at term

**DOI:** 10.1007/s00404-025-08042-2

**Published:** 2025-04-29

**Authors:** Alex Horky, Marita Wasenitz, Carlotta Iacovella, Franz Bahlmann, Ammar Al Naimi

**Affiliations:** 1 Department of Obstetrics and Gynecology, Buergerhospital - Dr. Senckenberg Foundation, Nibelungenallee 37-41, 60318 Frankfurt, Hessen Germany; 2 Department of Obstetrics and Prenatal Medicine, Goethe University, University Hospital of Frankfurt, Hessen, Germany

**Keywords:** Artificial intelligence, Sonography, Fetal weight assessment, Sonographer’s experience

## Abstract

**Purpose:**

The aim of this study is to investigate the differences in the accuracy of sonographic antenatal fetal weight estimation at term with artificial intelligence (AI) compared to that of clinical sonographers at different levels of experience.

**Methods:**

This is a prospective cohort study where pregnant women at term scheduled for an imminent elective cesarean section were recruited. Three independent antenatal fetal weight estimations for each fetus were blindly measured by an experienced resident physician with level I qualification from the German Society for Ultrasound in Medicine (group 1), a senior physician with level II qualification (group 2), and an AI-supported algorithm (group 3) using Hadlock formula 3. The differences between the three groups and the actual birth weight were examined with a paired *t*-test. A variation within 10% of birth weight was deemed accurate, and the diagnostic accuracies of both groups 1 and 3 compared to group 2 were assessed using receiver operating characteristic (ROC) curves. The association between accuracy and potential influencing factors including gestational age, fetal position, maternal age, maternal body mass index (BMI), twins, neonatal gender, placental position, gestational diabetes, and amniotic fluid index was tested with univariate logistic regression. A sensitivity analysis by inflating the estimated weights by daily 25 grams (g) gain for days between examination and birth was conducted.

**Results:**

300 fetuses at a mean gestational week of 38.7 ± 1.1 were included in this study and examined on median 2 (2–4) days prior to delivery. Average birth weight was 3264.6 ± 530.7 g and the mean difference of the sonographic estimated fetal weight compared to birthweight was −203.6 ± 325.4 g, −132.2 ± 294.1 g, and −338.4 ± 606.2 g for groups 1, 2, and 3 respectively. The estimated weight was accurate in 62% (56.2%, 67.5%), 70% (64.5%, 75,1%), and 48.3% (42.6%, 54.1%) for groups 1, 2, and 3 respectively. The diagnostic accuracy measures for groups 1 and 3 compared to group 2 resulted in 55.7% (48.7%, 62.5%) and 68.6% (61.8%, 74.8%) sensitivity, 68.9% (58.3%, 78.2%) and 53.3% (42.5%, 63.9%) specificity and 0.62 (0.56, 0.68) and 0.61 (0.55, 0.67) area under the ROC curves respectively. There was no association between accuracy and the investigated variables. Adjusting for sensitivity analysis increased the accuracy to 68% (62.4%, 73.2%), 75% (69.7%, 79.8%), and 51.3% (45.5%, 57.1%), and changed the mean difference compared to birth weight to −136.1 ± 321.8 g, −64.7 ± 291.2 g, and −270.7 ± 605.2 g for groups 1, 2, and 3 respectively.

**Conclusion:**

The antenatal weight estimation by experienced specialists with high-level qualifications remains the gold standard and provides the highest precision. Nevertheless, the accuracy of this standard is less than 80% even after adjusting for daily weight gain. The tested AI-supported method exhibits high variability and requires optimization and validation before being reliably used in clinical practice.

## What does this study add to the clinical work

**Table:** 

The tested AI method is not valid for estimating fetal weight at term and adjustments are required before it replaces the gold standard estimations by experienced specialists with high-level qualifications.	

## Introduction

Fetal growth reflects the intrauterine health of the fetus and is thus a key predictor of perinatal outcomes. Deviation from physiological growth trajectories, both in terms of growth restriction (FGR) or as excessive growth large for gestational age (LGA), is associated with increased cardiovascular, metabolic, and perinatal risks [1–4]. Fetal weight is influenced by a multitude of factors including genetic and anthropometric characteristics, such as familial predisposition and ethnic background [5], as well as maternal conditions such as diabetes or hypertension [6, 7]. Accurate prenatal weight estimation is essential for identifying abnormalities, initiating therapeutic interventions, optimizing management of birth, and improving both fetal and maternal outcomes.

Ultrasound represents the gold standard for fetal weight estimation and relies primarily on biometric measurements, such as the head circumference, the abdominal circumference, and the length of the femur. The Hadlock formula combines these three measurements to derive an estimated fetal weight [3, 8]. However, external factors including maternal body mass index (BMI), fetal position, amniotic fluid volume, and experience level of the examiner can affect the accuracy of these sonographic measurements and subsequently the reliability of the estimated weight [9–11]. The application of artificial intelligence (AI) has gained increasing significance in the medical field [12–15]. AI-based systems have the potential to enhance the objectivity and the precision of sonographic fetal weight estimation, thereby minimizing subjective errors and improving clinical outcomes.

We hypothesize that AI-assisted weight estimation is as accurate as the estimation of experienced sonographic experts, and that it could be resistant to external influencing factors. The main aim of this study is to evaluate the accuracy of sonographic antenatal fetal weight estimation with AI compared to sonographers at novice and expert levels of experience. Moreover, we aim to examine the association of potential influencing factors with the accuracy of estimated weights.

## Methods

This is a prospective observational cohort study at a tertiary prenatal center where pregnant women with singletons or twins at term scheduled for an imminent elective cesarean section between 1 May and 31 December 2024 were recruited. Inclusion criteria were age over 18 years, informed consent and a planned cesarean section within 1–4 days. Women with lack of consent, onset of labor, rupture of membranes, and known fetal malformations were excluded.

Recruitment took place during standardized preoperative consultation dates and three independent antenatal fetal weight estimations for each fetus were measured by an experienced resident physician with level I qualification from the German Society for Ultrasound in Medicine (DEGUM) (group 1), a senior physician with level II qualification (group 2), and an AI-supported algorithm (group 3) using Hadlock formula 3 [16]. Examiners were unaware of the gestational age, the acquired measures, and the estimated fetal weight to reduce bias. GE Voluson E22 system equipped with an integrated AI-assisted software was utilized for the acquisition of both the manual measurements and the automated AI-assisted measurements. The required anatomical parameters were the head circumference, the abdominal circumference, and the femur length, and all measurements were performed in accordance with the INTERGROWTH-21st Project [17–19] criteria. Data collection was conducted blinded to the gestational age.

The descriptive statistics included either mean and standard deviation or median and interquartile range (IQR) for continuous variables, and frequency and proportion for categorical variables. The differences between the three groups and the actual birth weight were examined with a paired t-test. A variation within 10% of birth weight was deemed accurate, and the diagnostic accuracies of both groups 1 and 3 compared to group 2 were assessed using receiver operating characteristic (ROC) curves. The associations between accuracy and potential influencing factors including gestational age, fetal position, maternal age, maternal body mass index (BMI), twins, neonatal gender, placental position, gestational diabetes, and amniotic fluid index were tested with univariate logistic regression. A sensitivity analysis by inflating the estimated weights with 25 grams (g) per day gain for the time between examination and birth was conducted [20–22]. A secondary post hoc sensitivity analysis was conducted by analyzing 266 observations after excluding twins.

Stata^®^ (ver. 18, Texas, USA) was used to perform all statistical analyses, with p-value of 0.05 as a cut-off threshold for statistical significance and utilizing the 95% confidence intervals. All prenatal ultrasound findings and postnatal data, such as gestational age at delivery, birthweight, body length, and head circumference, were anonymized and processed in accordance with applicable data protection regulations. This study was approved by the Ethics Committee at the Hesse State Chamber of Physicians with reference number 2023-3526-evBO.

## Results

300 fetuses at a mean gestational week of 38.7 ± 1.1 were included in this study and examined on median 2 (2–4) days prior to delivery. The fetal position was cephalic in 234 (78 %) cases, 34 (11.3 %) observations were from twins, and 137 (45.7%) neonates were females. Maternal age was 34.9 ± 5.2 years and the maternal pre-gestational BMI was 25.4 ± 5.7. The mean birth weight was 3264.6 ± 530.7 g. The demographic characteristics of the study cohort are summarized in Table 1.

**Table 1:** A summary of the study cohort demographic characteristics

Characteristic	*N* (%) Mean ± SD Median (IQR)
Fetal Position: • Cephalic presentation • Breech presentation • Transverse presentation	234 (78) 47 (15.7) 19 (6.3)
Maternal age	34.9 ± 5.2
Twin fetuses	34 (11.3)
Neonatal female gender	137 (45.7)
Anterior Placenta	152 (50.7)
Gestational Week	38.7 ± 1.1
Pregestational BMI	25.4 ± 5.7
Days from examination to delivery	2 (2, 4)
Maximum single pocket of amniotic fluid (mm)	45 (35, 63)
Birth weight (g)	3264.6 ± 530.7
Head circumference at birth (cm)	37.1 ± 5.7
Length at birth (cm)	48.9 ± 5.9

*SD* standard deviation, *IQR* interquartile range, *BMI* body mass index

**Fig. 1:**
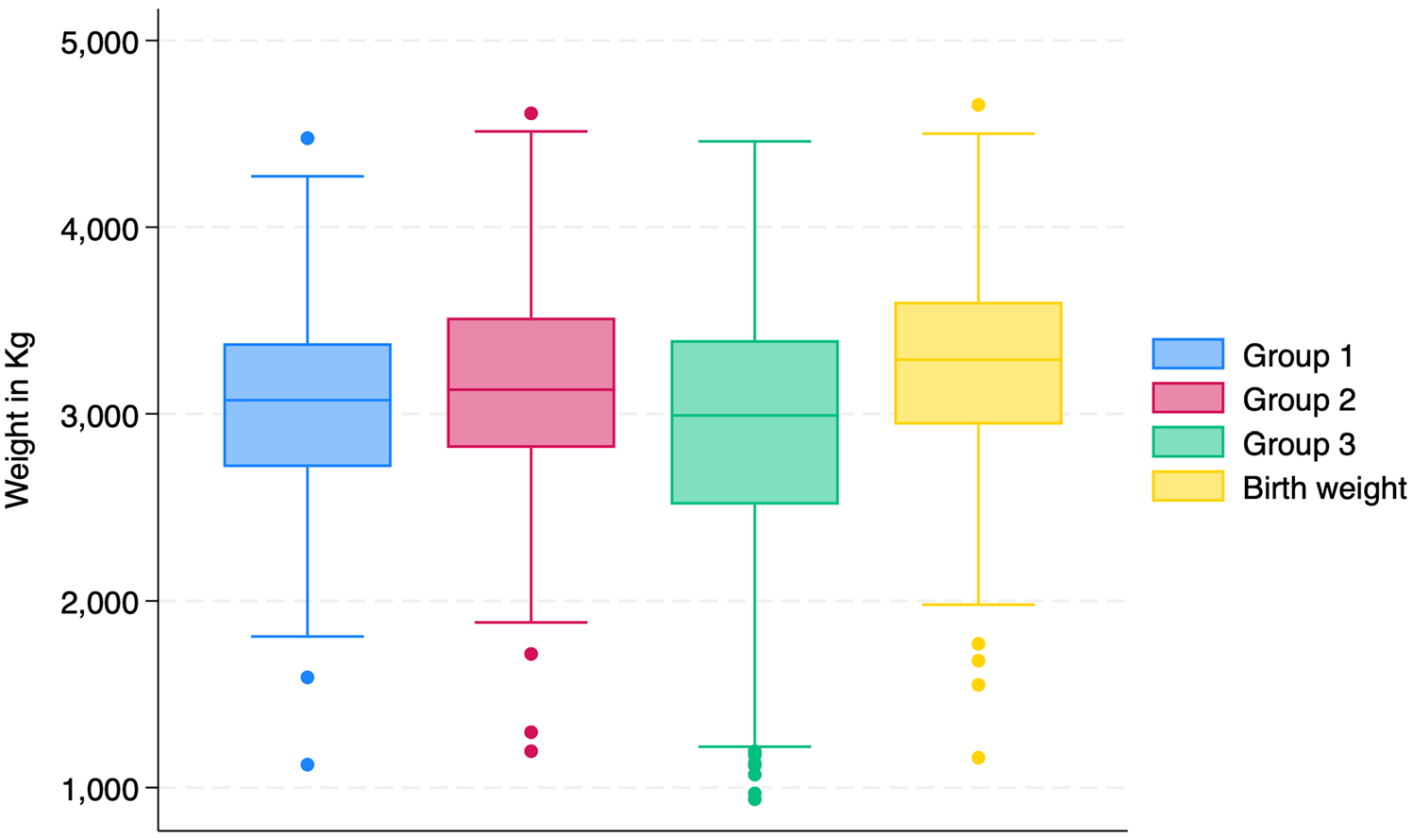
Boxplots of the actual birth weight and the estimated weights for groups 1, 2, and 3 in kilograms (kg)

The mean estimated fetal weight was 3061 ± 506.8 g for group 1, 3132.4 ± 534.8 g for group 2, and 2921.9 ± 713.4 g for group 3. All groups underestimated fetal weight, and the difference of the sonographic estimated fetal weight compared to birthweight was −203.6 ± 325.4 g, −132.2 ± 294.1 g, and −338.4 ± 606.2 g for groups 1,2, and 3 respectively (Fig. 1).

The estimated weight was accurate in 62% (56.2%, 67.5%), 70% (64.5%, 75.1%), and 48.3% (42.6%, 54.1%) for groups 1, 2, and 3 respectively. The diagnostic accuracy measures for groups 1 and 3 compared to group 2 resulted in 55.7% (48.7%, 62.5%) and 68.6% (61.8%, 74.8%) sensitivity, 68.9% (58.3%, 78.2%) and 53.3% (42.5%, 63.9%) specificity and 0.62 (0.56, 0.68) and 0.61 (0.55, 0.67) area under the ROC curves respectively.

None of the tested variables, including gestational age, cephalic position, maternal age, twins, BMI, anterior placenta, neonatal gender, gestational diabetes and amniotic fluid index showed an association with the odds ratio of an accurate sonographic weight estimation. The results of these logistic regression analyses are summarized in Table 2.

**Table 2:** The univariate logistic regression analysis for the association between different variables and odds of an accurate antenatal fetal weight estimation

Variable	Odds ratio for accurate antenatal weight estimate (95% CI)		
	Group 1	Group 2	Group 3
Gestational age (weeks)	0.92 (0.73, 1.16)	1.008 (0.79, 1.29)	1.09 (0.88, 1.34)
Cephalic position	1.03 (0.52, 2.01)	1.25 (0.58, 2.66)	0.56 (0.29, 1.06)
Maternal age (years)	1.01 (0.96, 1.05)	1.01 (0.97, 1.06)	0.97 (0.93, 1.01)
Neonatal female gender	1.27 (0.78, 2.07)	1.26 (0.74, 2.15)	1.04 (0.66, 1.63)
Twins	0.85 (0.40, 1.79)	0.57 (0.27, 1.21)	0.94 (0.46, 1.92)
Body mass index	0.99 (0.95, 1.03)	0.98 (0.94, 1.03)	1.002 (0.96, 1.04)
Anterior placenta	1.41 (0.87, 2.30)	1.78 (0.97, 3.02)	0.95 (0.60, 1.49)
Gestational diabetes	1.11 (0.54, 2.30)	2.05 (0.82, 5.09)	0.84 (0.43, 1.63)
Amniotic fluid index	1.0004 (0.99, 1.01)	1.01 (0.99, 1.03)	1.004 (0.99, 1.02)

*CI* confidence interval

**Fig. 2:**
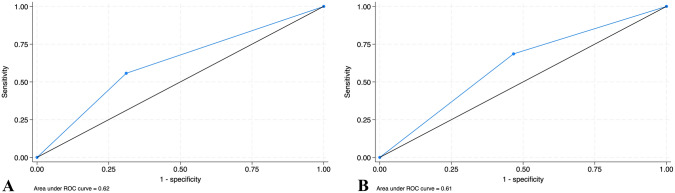
ROC curves for the estimated weight accuracy of **A** group 1 and **B** group 3 compared to group 2. *ROC* Receiver operator characteristic

Adjusting for sensitivity analysis increased the accuracy to 68% (62.4%, 73.2%), 75% (69.7%, 79.8%), and 51.3% (45.5%, 57.1%), and changed the mean difference compared to birth weight to −136.1 ± 321.8 g, −64.7 ± 291.2 g, and −270.7 ± 605.2 g for groups 1,2, and 3 respectively (Fig. 2).

The post hoc sensitivity analysis, after dropping the observations from twins, resulted in average birth weight of 3353.8 ± 465.6 g. This analysis led to the exclusion of few low-weight outliers from selectively growth restricted neonates in dichorionic pregnancies. The mean difference of the sonographic estimated fetal weight compared to birthweight was−209.3 ± 321.7 g, −128.4 ± 301.9 g, and −337.8 ± 622.5 g for groups 1, 2, and 3 respectively. The estimated weight was accurate in 61.3% (55.1%, 67.2%), 71.1% (65.2%, 76.4%), and 48.9% (42.7%, 55%) for groups 1, 2, and 3 respectively. The diagnostic accuracy measures for groups 1 and 3 compared to group 2 resulted in 67.2% (60.0%, 73.8%) and 55.0% (47.6%, 62.3%) sensitivity, 53.2% (41.5%, 64.7%) and 66.2% (54.6%, 76.6%) specificity and 0.60 (0.54, 0.67) and 0.61 (0.54, 0.67) area under the ROC curves respectively. Therefore, the results of this sensitivity analysis do not significantly deviate from the results of the main analysis that included twins.

## Discussion

This study shows that the accuracy of antenatal fetal weight estimation is significantly dependent on the sonographic operator for each of the examined three groups. However, irrespective of operator, all utilized measurement methods systematically underestimated the actual birth weight even after inflating for daily weight gain. Among the groups, the experienced senior physicians (Group 2) demonstrated the lowest underestimation and consequently the highest precision. Our findings align with a study conducted by Yau et al., which demonstrated that the accuracy of fetal weight prediction improves with the sonographic expertise. Similar to our work, they defined an accurate prediction as a difference of less than 10% of the actual birth weight. The frequency of accurate measurements was higher among specialists compared to less-experienced juniors [10]. These findings highlight the crucial role of advanced training and clinical experience in reaching accurate prenatal sonographic weight estimations. Moreover, consistent higher precision of experienced sonographers indicates the importance of education and structured training programs in obstetrical ultrasound. These could increase the collective experience among practitioners, thereby reduce discrepancies in estimated fetal weight and improve clinical decision-making and perinatal outcomes.

Rigorous sonographic training programs equip physicians with theoretical knowledge and practical skills necessary for enhancing clinical judgment and accurately assessing subtle physiological and pathological findings. Acquiring a DEGUM level II certification requires substantial clinical experience gained through multi-stage training. These clinicians possess extensive sonographic experience in recognizing normal physiological parameters in addition to identifying fetal malformations, pathological findings, or fetal growth restriction, and accordingly counseling involved patients [23, 24]. This is why group 2, with level II qualifications was more accurate and considered the gold standard for determining the diagnostic accuracy of the other groups.

The inaccuracy of a sonographic measure could be attributed to potentially limited visibility of the fetus at term as well as non-optimal position. There is a significant difference between the fetal position at term compared to the earlier weeks of gestation. This change is responsible for better accuracy of ellipse function measurements than distance function measurements [25]. Comparative studies showed that methods utilizing directly measured head and abdominal circumferences provided significantly more accurate estimates than those relying on diameter-based calculations [25]. Aside from the used measurements, the utilized formula affects the fetal weight estimation, and various formulas have been proposed. Hadlock’s three-parameter formula based on head circumference, abdominal circumference, and femur length is considered the gold standard formula [8]. Both Hammami et al. and Nicolaides et al. have shown that substituting the biparietal diameter for the head circumference reduces accuracy, which is a conclusion propagated by the Intergrowth 21st Study [16, 18]. This is the reason why we opted to utilize the Hadlock formula 3 and the ellipse function to achieve the highest possible accuracy.

**Fig. 3:**
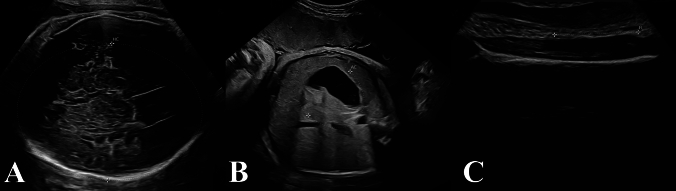
Ultrasound images showing wrong AI measurements leading to underestimation of **A** the head circumference, **B** the abdominal circumference, and **C** the femur length

Arryoyo et al. showed that AI resulted in higher mean negative error rate compared to human measurement, and it was higher for head circumference 7.9% than biparietal distance 5.6% [26]. Nevertheless, our utilized AI model in group 3 frequently failed to identify the correct biometrical measurements as shown in Figure 3 which subsequently led to its inaccuracy and weight underestimation.

Plotka et al. showed that AI-based methods are equivalent to operator-driven methods in estimating weights from sonographic video sequences [12]. These findings cannot be directly compared to our study. One reason is that Plotka et al. utilized an AI model trained by a dataset from their own cohort. Moreover, their study population included a larger range of gestational ages. Furthermore, their accuracy definition was based on manual estimation instead of the actual birth weight as in our study.

Further AI software development through deep learning could significantly improve image differentiation in prenatal ultrasound diagnostics. Deep learning algorithms, particularly convolutional neural networks, have the potential to increase the accuracy of image analysis by automating and optimizing segmentation and contour detection of fetal structures. Compared to traditional image processing methods, deep learning models can recognize deeper and more complex patterns in the ultrasound images, resulting in more precise identification of tissues and organs that may be more difficult to differentiate using traditional methods [27]. Deep learning algorithms uncover intricate patterns from imaging data; thus, they significantly outperform traditional image processing techniques in both accuracy and efficiency [28]. Deep learning models trained on even larger datasets of ultrasound images could overcome these challenges by detecting the variations in the image data and automatically adjusting them to enable more precise analysis. Continuous improvement of the algorithms, using larger, more diverse datasets, and deep learning could allow for the detection and correction of sonographic artifacts. This would increase the accuracy of fetal measurements and lead to a more reliable estimate of birth weight and better clinical decisions [29, 30]. However, it is essential to ensure a responsible and transparent use of AI systems, particularly with respect to liability concerns and the interpretability of resultsKlicken oder tippen Sie hier, um Text einzugeben.

None of the examined potential influencing factors in this study, including gestational age, fetal position, maternal age, BMI, multiple pregnancy, placental location, or maternal gestational diabetes, was associated with the precision of the weight estimation. Ultrasound artifacts combined with disruption of the fetal skull by the normal cranial sutures result in image differentiation and identification difficulties in the third trimester. However, these do not result in relevant percentage differences between examinations during second and third trimesters [31].

The sensitivity analysis, which adjusted the estimated fetal weight by 25 g per day, further emphasizes that accounting for the time interval between the ultrasound examination and delivery leads to a significant improvement in measurement accuracy. Accurate weight estimation significantly affects the perinatal outcomes. Underestimation increases the risk of obstructed labor, difficult cesarean section, and shoulder dystocia [32–34]. Reliable fetal weight assessment is essential for optimizing management strategies and promoting safe perinatal care.

This study has several strengths including the prospective study design, blinding to gestational age, utilizing appropriate statistical methods, and including three comparative cohorts. Nevertheless, weaknesses, such as narrow range of examined gestational age, inapplicability for fetuses with malformations, and limited information about the intricacies of the AI model, affect the generalizability and external validity of our findings. Including twins in this study could be seen as a limitation due to the relatively complex fetal position and its impact on AI’s precision. Nevertheless, we believe that including twins increased generalizability and allowed for examining the effect of this potential confounder on obtained accuracy. Moreover, a secondary post hoc sensitivity analysis after excluding observations from twins did not change the main outcomes of the study.

To conclude, the findings of this study show that antenatal weight estimation by experienced specialists with high level qualifications remains the gold standard and provides the highest precision. Nevertheless, the accuracy of this standard is less than 80% even after adjusting for daily weight gain. The tested AI-supported method exhibits high variability and requires optimization and validation before being reliably used in clinical practice. Therefore, this provides valuable insights for improving diagnostic precision and patient safety in prenatal ultrasound diagnostics.

## Data Availability

The datasets used and/or analysed during the current study are available from the corresponding author on reasonable request.

## Code availability

Non-applicable.
